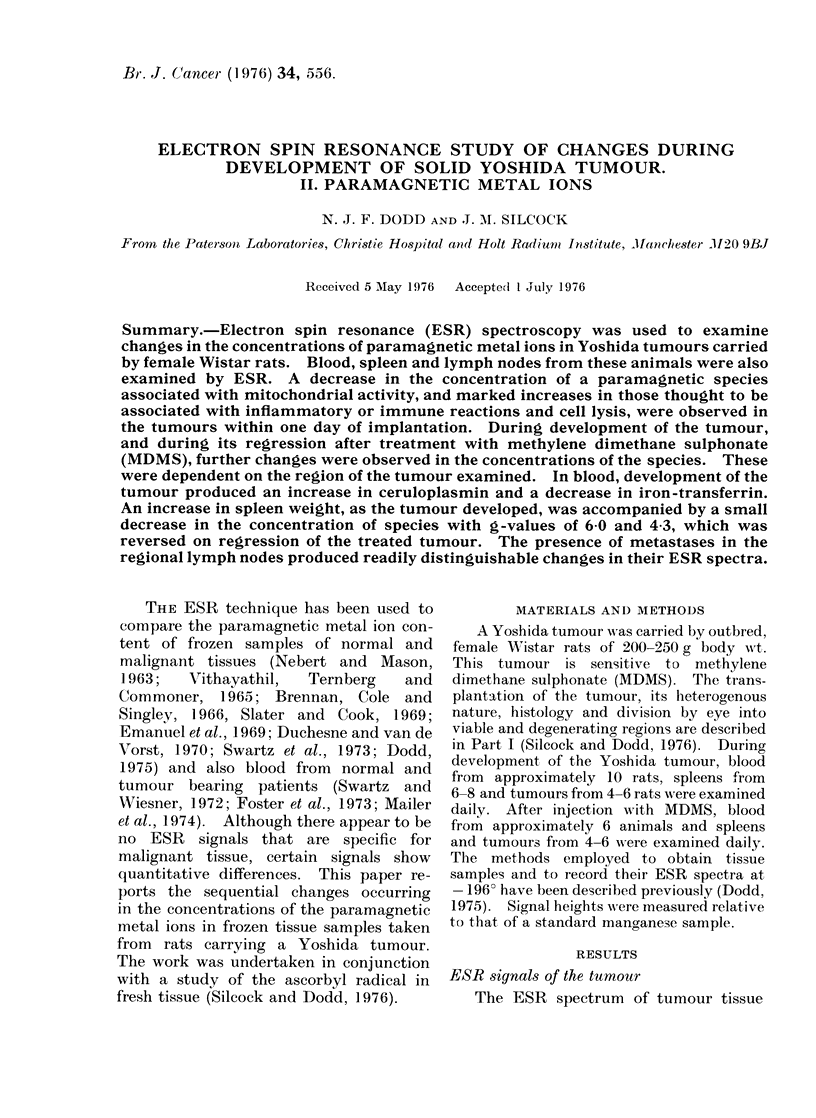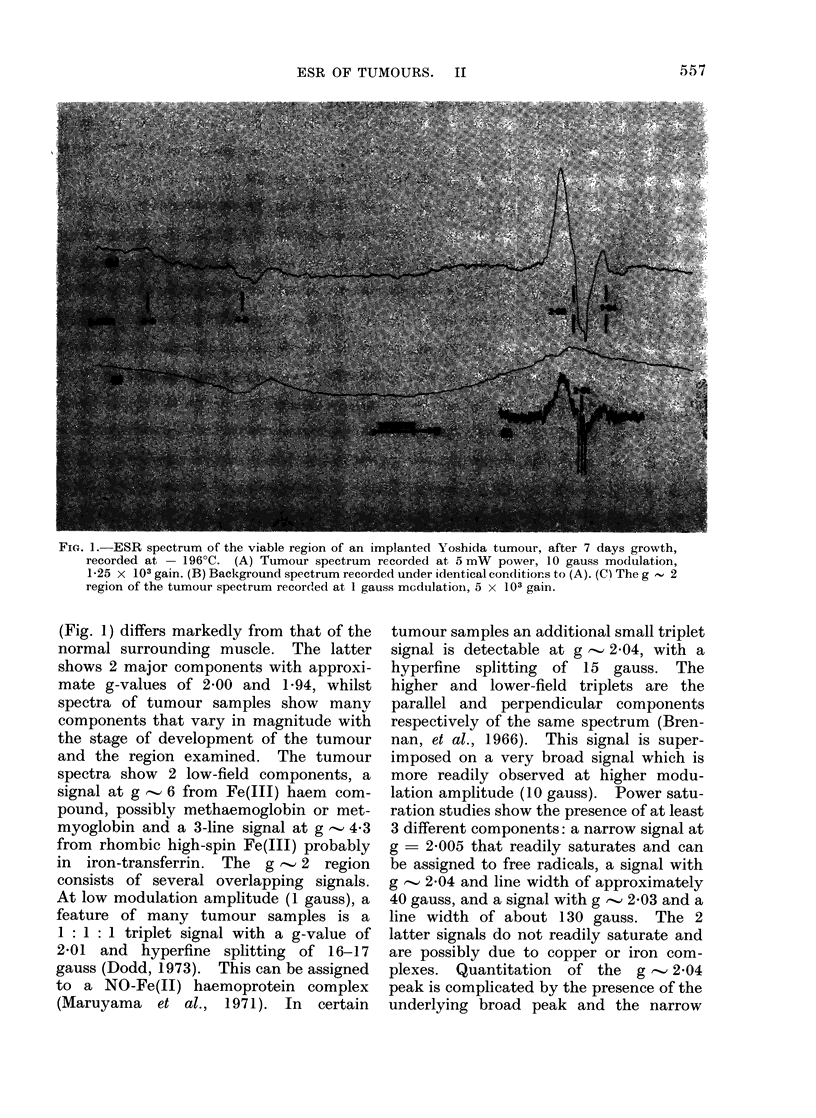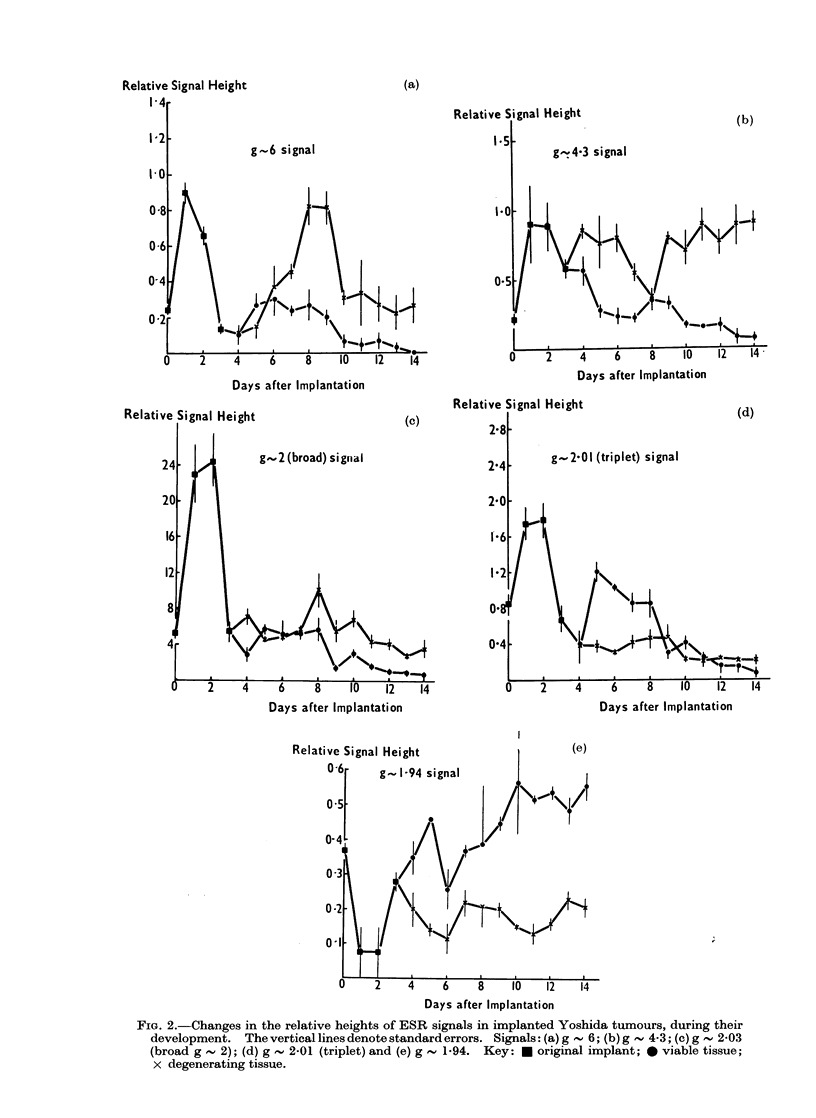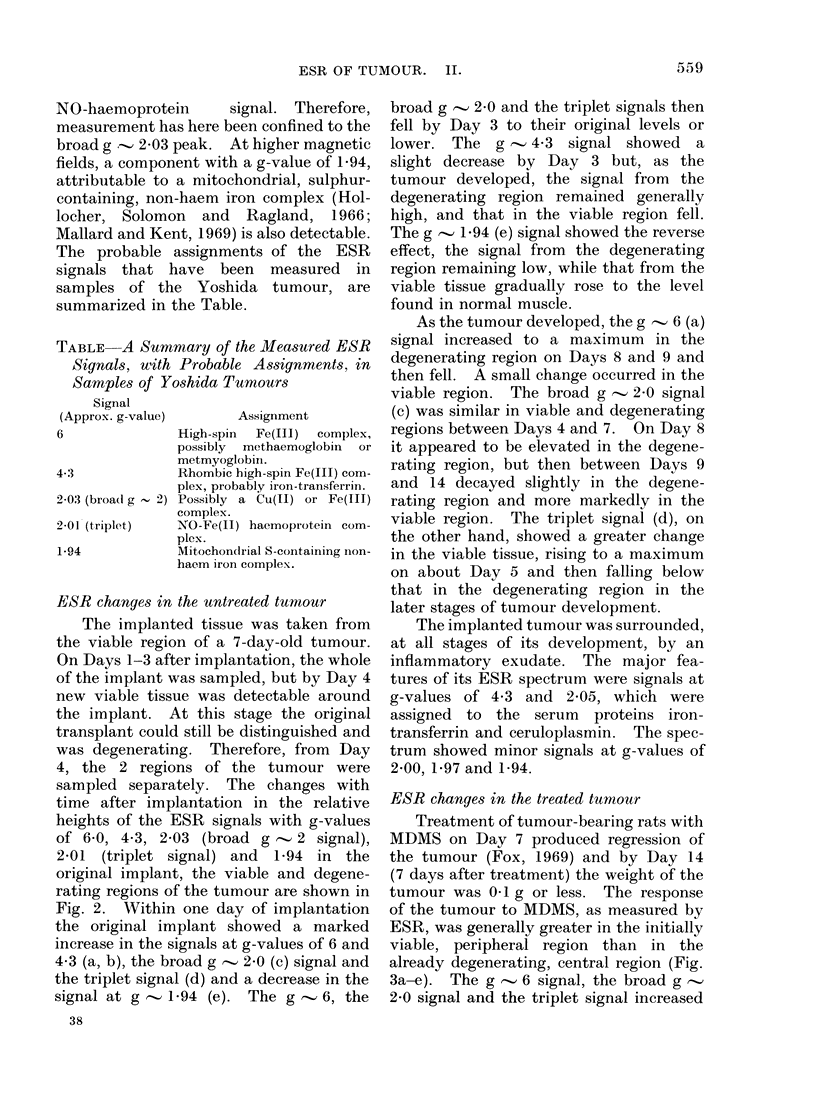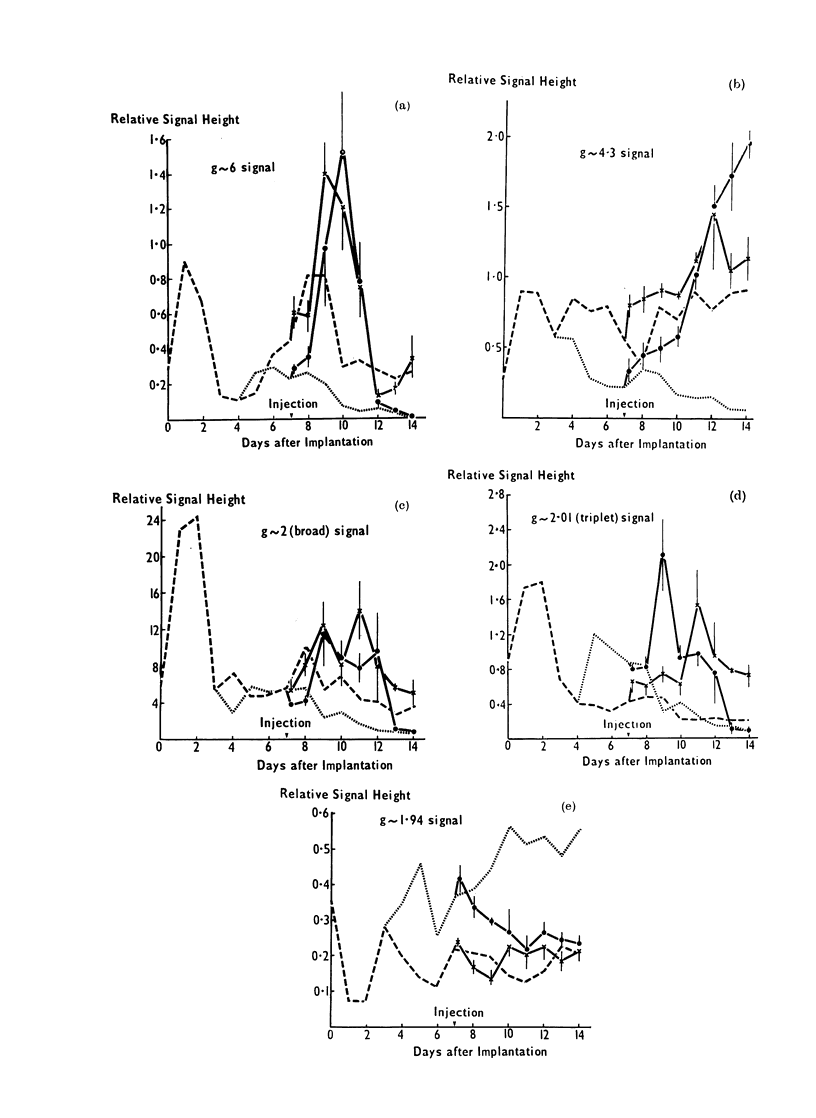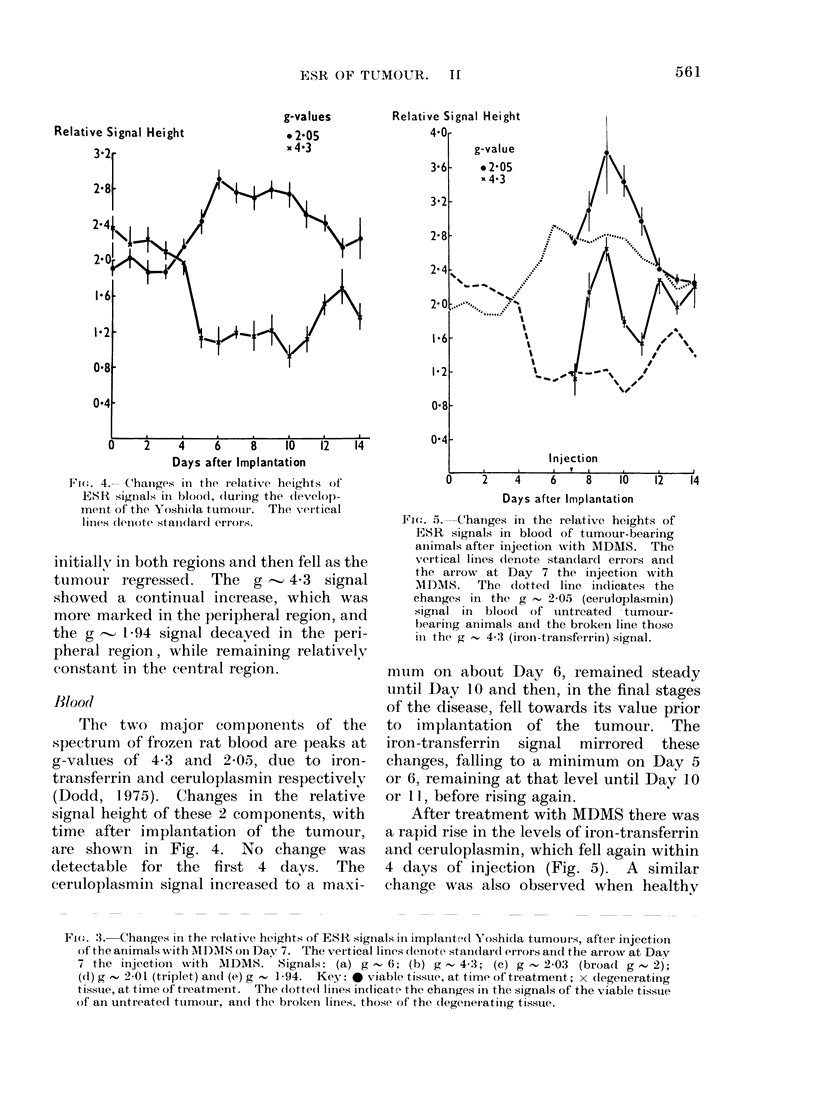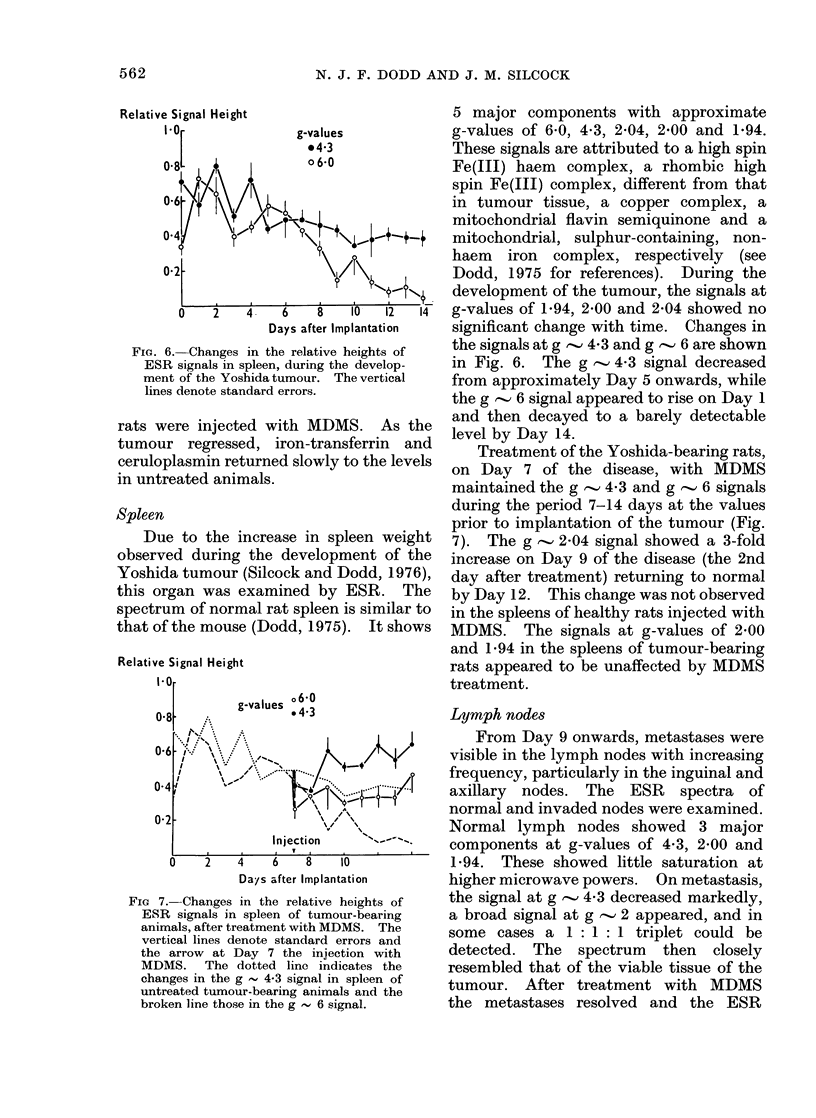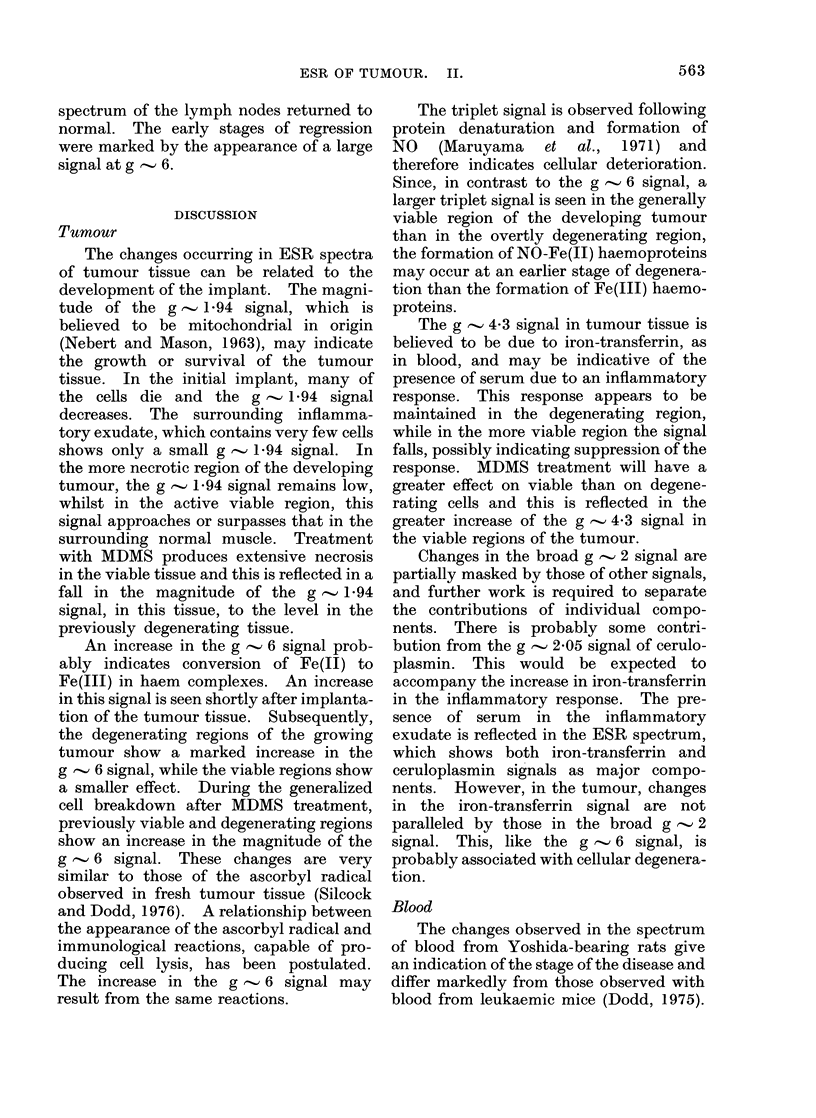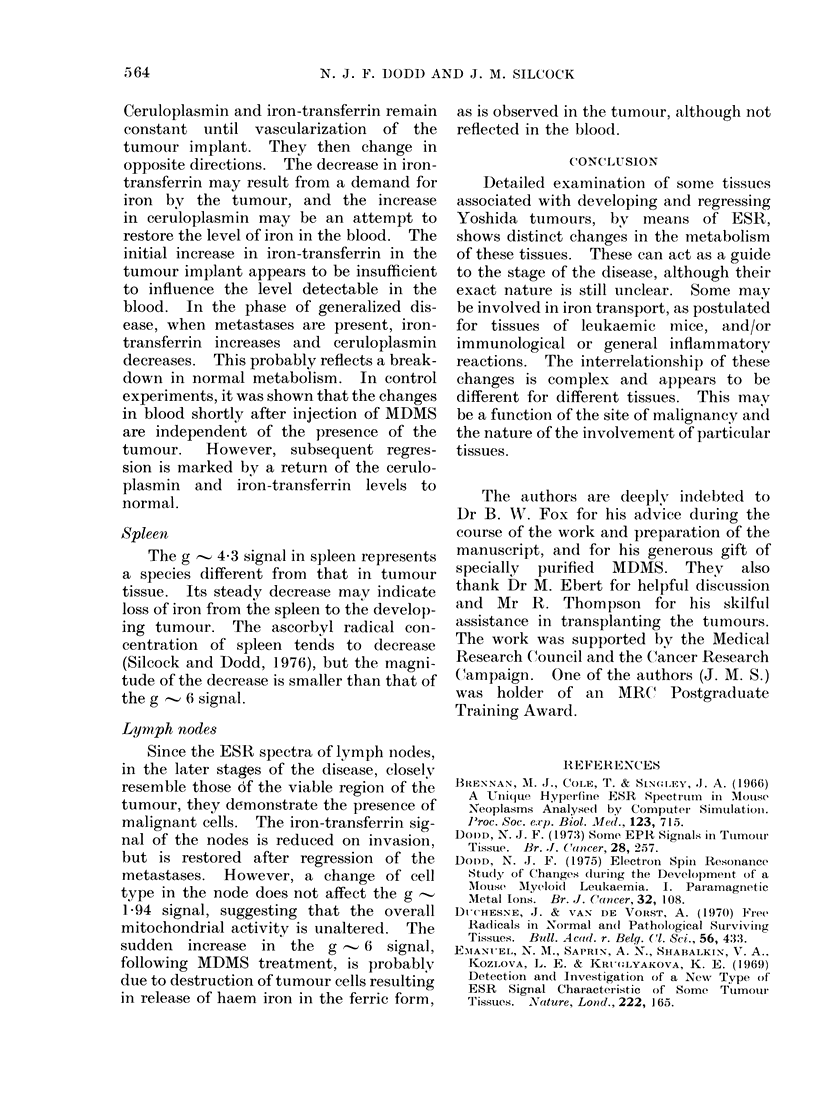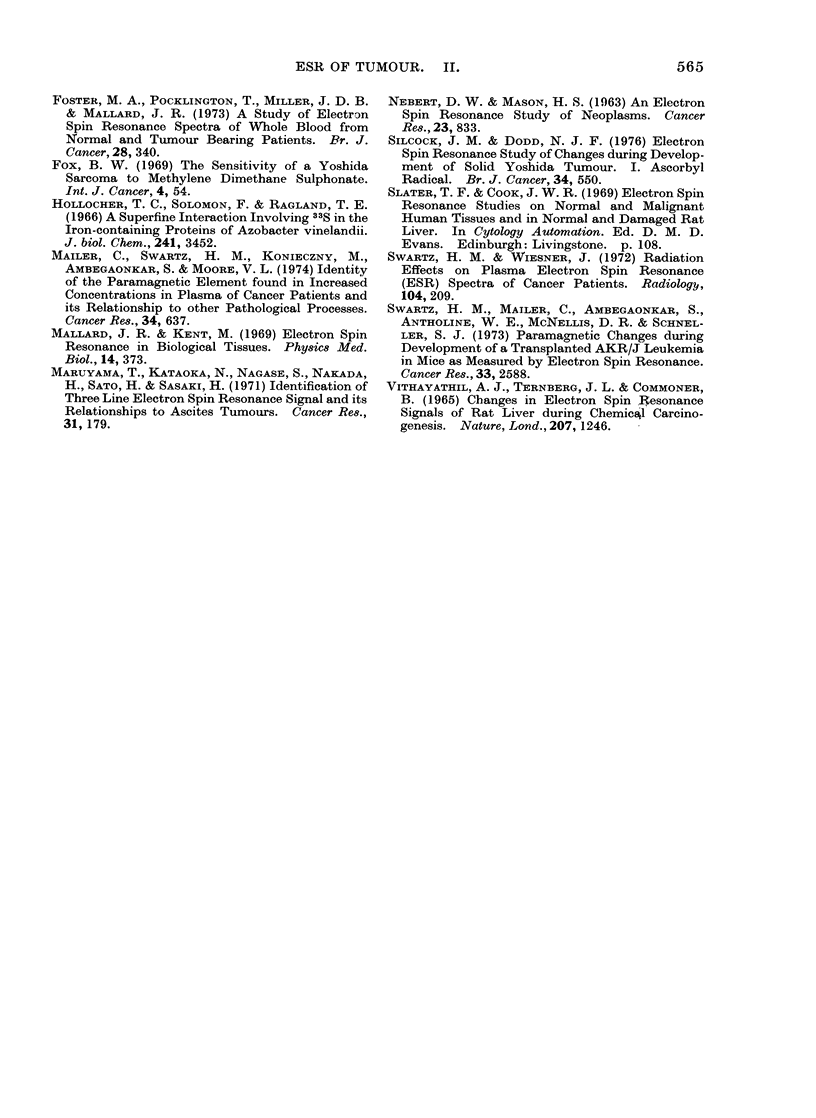# Electron spin resonance study of changes during development of solid yoshida tumour. II. Paramagnetic metal ions.

**DOI:** 10.1038/bjc.1976.211

**Published:** 1976-11

**Authors:** N. J. Dodd, J. M. Silcock

## Abstract

**Images:**


*Br. J. Cancer* (1976) **34,** 556.

### ELECTRON SPIN RESONANCE STUDY OF CHANGES DURING DEVELOPMENT OF SOLID YOSHIDA TUMOUR. II. PARAMAGNETIC METAL IONS

N. J. F. DODD AND J. M. SILCOCK

*From the Paterson Laboratories, Christie Hospital and Holt Radium Institute, Manchester M20 9BJ*

Received 5 May 1976 Accepted 1 July 1976

**Summary.—Electron spin resonance (ESR) spectroscopy was used to examine changes in the concentrations of paramagnetic metal ions in Yoshida tumours carried by female Wistar rats. Blood, spleen and lymph nodes from these animals were also examined by ESR. A decrease in the concentration of a paramagnetic species associated with mitochondrial activity, and marked increases in those thought to be associated with inflammatory or immune reactions and cell lysis, were observed in the tumours within one day of implantation. During development of the tumour, and during its regression after treatment with methylene dimethane sulphonate (MDMS), further changes were observed in the concentrations of the species. These were dependent on the region of the tumour examined. In blood, development of the tumour produced an increase in ceruloplasmin and a decrease in iron-transferrin. An increase in spleen weight, as the tumour developed, was accompanied by a small decrease in the concentration of species with g-values of 6·0 and 4·3, which was reversed on regression of the treated tumour. The presence of metastases in the regional lymph nodes produced readily distinguishable changes in their ESR spectra.**

THE ESR technique has been used to compare the paramagnetic metal ion content of frozen samples of normal and malignant tissues (Nebert and Mason, 1963; Vithayathil, Ternberg and Commoner, 1965; Brennan, Cole and Singley, 1966, Slater and Cook, 1969; Emanuel *et al.*, 1969; Duchesne and van de Vorst, 1970; Swartz *et al.*, 1973; Dodd, 1975) and also blood from normal and tumour bearing patients (Swartz and Wiesner, 1972; Foster *et al.*, 1973; Mailer *et al.*, 1974). Although there appear to be no ESR signals that are specific for malignant tissue, certain signals show quantitative differences. This paper reports the sequential changes occurring in the concentrations of the paramagnetic metal ions in frozen tissue samples taken from rats carrying a Yoshida tumour. The work was undertaken in conjunction with a study of the ascorbyl radical in fresh tissue (Silcock and Dodd, 1976).

### MATERIALS AND METHODS

A Yoshida tumour was carried by outbred, female Wistar rats of 200–250 g body wt. This tumour is sensitive to methylene dimethane sulphonate (MDMS). The transplantation of the tumour, its heterogenous nature, histology and division by eye into viable and degenerating regions are described in Part I (Silcock and Dodd, 1976). During development of the Yoshida tumour, blood from approximately 10 rats, spleens from 6–8 and tumours from 4–6 rats were examined daily. After injection with MDMS, blood from approximately 6 animals and spleens and tumours from 4–6 were examined daily. The methods employed to obtain tissue samples and to record their ESR spectra at $-196^{\circ}$ have been described previously (Dodd, 1975). Signal heights were measured relative to that of a standard manganese sample.

### RESULTS

### *ESR signals of the tumour*

The ESR spectrum of tumour tissue

FIG. 1.—ESR spectrum of the viable region of an implanted Yoshida tumour, after 7 days growth, recorded at − 196°C. (A) Tumour spectrum recorded at 5 mW power, 10 gauss modulation, $1\cdot25 \times 10^3$ gain. (B) Background spectrum recorded under identical conditions to (A). (C) The g ~ 2 region of the tumour spectrum recorded at 1 gauss modulation, $5 \times 10^3$ gain.

(Fig. 1) differs markedly from that of the normal surrounding muscle. The latter shows 2 major components with approximate g-values of 2·00 and 1·94, whilst spectra of tumour samples show many components that vary in magnitude with the stage of development of the tumour and the region examined. The tumour spectra show 2 low-field components, a signal at g ~ 6 from Fe(III) haem compound, possibly methaemoglobin or metmyoglobin and a 3-line signal at g ~ 4·3 from rhombic high-spin Fe(III) probably in iron-transferrin. The g ~ 2 region consists of several overlapping signals. At low modulation amplitude (1 gauss), a feature of many tumour samples is a 1 : 1 : 1 triplet signal with a g-value of 2·01 and hyperfine splitting of 16–17 gauss (Dodd, 1973). This can be assigned to a NO-Fe(II) haemoprotein complex (Maruyama *et al.*, 1971). In certain tumour samples an additional small triplet signal is detectable at g ~ 2·04, with a hyperfine splitting of 15 gauss. The higher and lower-field triplets are the parallel and perpendicular components respectively of the same spectrum (Brennan, *et al.*, 1966). This signal is superimposed on a very broad signal which is more readily observed at higher modulation amplitude (10 gauss). Power saturation studies show the presence of at least 3 different components: a narrow signal at g = 2·005 that readily saturates and can be assigned to free radicals, a signal with g ~ 2·04 and line width of approximately 40 gauss, and a signal with g ~ 2·03 and a line width of about 130 gauss. The 2 latter signals do not readily saturate and are possibly due to copper or iron complexes. Quantitation of the g ~ 2·04 peak is complicated by the presence of the underlying broad peak and the narrow

FIG. 2.—Changes in the relative heights of ESR signals in implanted Yoshida tumours, during their development. The vertical lines denote standard errors. Signals: (a) g ~ 6; (b) g ~ 4·3; (c) g ~ 2·03 (broad g ~ 2); (d) g ~ 2·01 (triplet) and (e) g ~ 1·94. Key: ■ original implant; ● viable tissue; × degenerating tissue.

NO-haemoprotein signal. Therefore, measurement has here been confined to the broad g $\sim$ 2·03 peak. At higher magnetic fields, a component with a g-value of 1·94, attributable to a mitochondrial, sulphur-containing, non-haem iron complex (Hollocher, Solomon and Ragland, 1966; Mallard and Kent, 1969) is also detectable. The probable assignments of the ESR signals that have been measured in samples of the Yoshida tumour, are summarized in the Table.

TABLE—*A Summary of the Measured ESR Signals, with Probable Assignments, in Samples of Yoshida Tumours*

| Signal (Approx. g-value) | Assignment |
|---|---|
| 6 | High-spin Fe(III) complex, possibly methaemoglobin or metmyoglobin. |
| 4·3 | Rhombic high-spin Fe(III) complex, probably iron-transferrin. |
| 2·03 (broad g $\sim$ 2) | Possibly a Cu(II) or Fe(III) complex. |
| 2·01 (triplet) | NO-Fe(II) haemoprotein complex. |
| 1·94 | Mitochondrial S-containing non-haem iron complex. |

*ESR changes in the untreated tumour*

The implanted tissue was taken from the viable region of a 7-day-old tumour. On Days 1–3 after implantation, the whole of the implant was sampled, but by Day 4 new viable tissue was detectable around the implant. At this stage the original transplant could still be distinguished and was degenerating. Therefore, from Day 4, the 2 regions of the tumour were sampled separately. The changes with time after implantation in the relative heights of the ESR signals with g-values of 6·0, 4·3, 2·03 (broad g $\sim$ 2 signal), 2·01 (triplet signal) and 1·94 in the original implant, the viable and degenerating regions of the tumour are shown in Fig. 2. Within one day of implantation the original implant showed a marked increase in the signals at g-values of 6 and 4·3 (a, b), the broad g $\sim$ 2·0 (c) signal and the triplet signal (d) and a decrease in the signal at g $\sim$ 1·94 (e). The g $\sim$ 6, the broad g $\sim$ 2·0 and the triplet signals then fell by Day 3 to their original levels or lower. The g $\sim$ 4·3 signal showed a slight decrease by Day 3 but, as the tumour developed, the signal from the degenerating region remained generally high, and that in the viable region fell. The g $\sim$ 1·94 (e) signal showed the reverse effect, the signal from the degenerating region remaining low, while that from the viable tissue gradually rose to the level found in normal muscle.

As the tumour developed, the g $\sim$ 6 (a) signal increased to a maximum in the degenerating region on Days 8 and 9 and then fell. A small change occurred in the viable region. The broad g $\sim$ 2·0 signal (c) was similar in viable and degenerating regions between Days 4 and 7. On Day 8 it appeared to be elevated in the degenerating region, but then between Days 9 and 14 decayed slightly in the degenerating region and more markedly in the viable region. The triplet signal (d), on the other hand, showed a greater change in the viable tissue, rising to a maximum on about Day 5 and then falling below that in the degenerating region in the later stages of tumour development.

The implanted tumour was surrounded, at all stages of its development, by an inflammatory exudate. The major features of its ESR spectrum were signals at g-values of 4·3 and 2·05, which were assigned to the serum proteins iron-transferrin and ceruloplasmin. The spectrum showed minor signals at g-values of 2·00, 1·97 and 1·94.

*ESR changes in the treated tumour*

Treatment of tumour-bearing rats with MDMS on Day 7 produced regression of the tumour (Fox, 1969) and by Day 14 (7 days after treatment) the weight of the tumour was 0·1 g or less. The response of the tumour to MDMS, as measured by ESR, was generally greater in the initially viable, peripheral region than in the already degenerating, central region (Fig. 3a–e). The g $\sim$ 6 signal, the broad g $\sim$ 2·0 signal and the triplet signal increased

(a)

Relative Signal Height g~6 signal

1·6

1·4

1·2

1·0

0·8

0·6

0·4

0·2

Injection

0

2

4

6

8

10

12

14

Days after Implantation

(b)

Relative Signal Height g~4·3 signal

2·0

1·5

1·0

0·5

Injection

2

4

6

8

10

12

14

Days after Implantation

(c)

Relative Signal Height g~2 (broad) signal

24

20

16

12

8

4

Injection

0

2

4

6

8

10

12

14

Days after Implantation

(d)

Relative Signal Height g~2·01 (triplet) signal

2·8

2·4

2·0

1·6

1·2

0·8

0·4

Injection

0

2

4

6

8

10

12

14

Days after Implantation

(e)

Relative Signal Height g~1·94 signal

0·6

0·5

0·4

0·3

0·2

0·1

Injection

0

2

4

6

8

10

12

14

Days after Implantation

FIG. 4.—Changes in the relative heights of ESR signals in blood, during the development of the Yoshida tumour. The vertical lines denote standard errors.

FIG. 5.—Changes in the relative heights of ESR signals in blood of tumour-bearing animals after injection with MDMS. The vertical lines denote standard errors and the arrow at Day 7 the injection with MDMS. The dotted line indicates the changes in the g ~ 2·05 (ceruloplasmin) signal in blood of untreated tumour-bearing animals and the broken line those in the g ~ 4·3 (iron-transferrin) signal.

initially in both regions and then fell as the tumour regressed. The g ~ 4·3 signal showed a continual increase, which was more marked in the peripheral region, and the g ~ 1·94 signal decayed in the peripheral region, while remaining relatively constant in the central region.

*Blood*

The two major components of the spectrum of frozen rat blood are peaks at g-values of 4·3 and 2·05, due to iron-transferrin and ceruloplasmin respectively (Dodd, 1975). Changes in the relative signal height of these 2 components, with time after implantation of the tumour, are shown in Fig. 4. No change was detectable for the first 4 days. The ceruloplasmin signal increased to a maximum on about Day 6, remained steady until Day 10 and then, in the final stages of the disease, fell towards its value prior to implantation of the tumour. The iron-transferrin signal mirrored these changes, falling to a minimum on Day 5 or 6, remaining at that level until Day 10 or 11, before rising again.

After treatment with MDMS there was a rapid rise in the levels of iron-transferrin and ceruloplasmin, which fell again within 4 days of injection (Fig. 5). A similar change was also observed when healthy

FIG. 3.—Changes in the relative heights of ESR signals in implanted Yoshida tumours, after injection of the animals with MDMS on Day 7. The vertical lines denote standard errors and the arrow at Day 7 the injection with MDMS. Signals: (a) g ~ 6; (b) g ~ 4·3; (c) g ~ 2·03 (broad g ~ 2); (d) g ~ 2·01 (triplet) and (e) g ~ 1·94. Key: ● viable tissue, at time of treatment; × degenerating tissue, at time of treatment. The dotted lines indicate the changes in the signals of the viable tissue of an untreated tumour, and the broken lines, those of the degenerating tissue.

FIG. 6.—Changes in the relative heights of ESR signals in spleen, during the development of the Yoshida tumour. The vertical lines denote standard errors.

rats were injected with MDMS. As the tumour regressed, iron-transferrin and ceruloplasmin returned slowly to the levels in untreated animals.

*Spleen*

Due to the increase in spleen weight observed during the development of the Yoshida tumour (Silcock and Dodd, 1976), this organ was examined by ESR. The spectrum of normal rat spleen is similar to that of the mouse (Dodd, 1975). It shows

FIG 7.—Changes in the relative heights of ESR signals in spleen of tumour-bearing animals, after treatment with MDMS. The vertical lines denote standard errors and the arrow at Day 7 the injection with MDMS. The dotted line indicates the changes in the g ~ 4·3 signal in spleen of untreated tumour-bearing animals and the broken line those in the g ~ 6 signal.

5 major components with approximate g-values of 6·0, 4·3, 2·04, 2·00 and 1·94. These signals are attributed to a high spin Fe(III) haem complex, a rhombic high spin Fe(III) complex, different from that in tumour tissue, a copper complex, a mitochondrial flavin semiquinone and a mitochondrial, sulphur-containing, non-haem iron complex, respectively (see Dodd, 1975 for references). During the development of the tumour, the signals at g-values of 1·94, 2·00 and 2·04 showed no significant change with time. Changes in the signals at g ~ 4·3 and g ~ 6 are shown in Fig. 6. The g ~ 4·3 signal decreased from approximately Day 5 onwards, while the g ~ 6 signal appeared to rise on Day 1 and then decayed to a barely detectable level by Day 14.

Treatment of the Yoshida-bearing rats, on Day 7 of the disease, with MDMS maintained the g ~ 4·3 and g ~ 6 signals during the period 7–14 days at the values prior to implantation of the tumour (Fig. 7). The g ~ 2·04 signal showed a 3-fold increase on Day 9 of the disease (the 2nd day after treatment) returning to normal by Day 12. This change was not observed in the spleens of healthy rats injected with MDMS. The signals at g-values of 2·00 and 1·94 in the spleens of tumour-bearing rats appeared to be unaffected by MDMS treatment.

*Lymph nodes*

From Day 9 onwards, metastases were visible in the lymph nodes with increasing frequency, particularly in the inguinal and axillary nodes. The ESR spectra of normal and invaded nodes were examined. Normal lymph nodes showed 3 major components at g-values of 4·3, 2·00 and 1·94. These showed little saturation at higher microwave powers. On metastasis, the signal at g ~ 4·3 decreased markedly, a broad signal at g ~ 2 appeared, and in some cases a 1 : 1 : 1 triplet could be detected. The spectrum then closely resembled that of the viable tissue of the tumour. After treatment with MDMS the metastases resolved and the ESR

spectrum of the lymph nodes returned to normal. The early stages of regression were marked by the appearance of a large signal at $g \sim 6$.

### DISCUSSION

### *Tumour*

The changes occurring in ESR spectra of tumour tissue can be related to the development of the implant. The magnitude of the $g \sim 1{\cdot}94$ signal, which is believed to be mitochondrial in origin (Nebert and Mason, 1963), may indicate the growth or survival of the tumour tissue. In the initial implant, many of the cells die and the $g \sim 1{\cdot}94$ signal decreases. The surrounding inflammatory exudate, which contains very few cells shows only a small $g \sim 1{\cdot}94$ signal. In the more necrotic region of the developing tumour, the $g \sim 1{\cdot}94$ signal remains low, whilst in the active viable region, this signal approaches or surpasses that in the surrounding normal muscle. Treatment with MDMS produces extensive necrosis in the viable tissue and this is reflected in a fall in the magnitude of the $g \sim 1{\cdot}94$ signal, in this tissue, to the level in the previously degenerating tissue.

An increase in the $g \sim 6$ signal probably indicates conversion of Fe(II) to Fe(III) in haem complexes. An increase in this signal is seen shortly after implantation of the tumour tissue. Subsequently, the degenerating regions of the growing tumour show a marked increase in the $g \sim 6$ signal, while the viable regions show a smaller effect. During the generalized cell breakdown after MDMS treatment, previously viable and degenerating regions show an increase in the magnitude of the $g \sim 6$ signal. These changes are very similar to those of the ascorbyl radical observed in fresh tumour tissue (Silcock and Dodd, 1976). A relationship between the appearance of the ascorbyl radical and immunological reactions, capable of producing cell lysis, has been postulated. The increase in the $g \sim 6$ signal may result from the same reactions.

The triplet signal is observed following protein denaturation and formation of NO (Maruyama *et al.*, 1971) and therefore indicates cellular deterioration. Since, in contrast to the $g \sim 6$ signal, a larger triplet signal is seen in the generally viable region of the developing tumour than in the overtly degenerating region, the formation of NO-Fe(II) haemoproteins may occur at an earlier stage of degeneration than the formation of Fe(III) haemoproteins.

The $g \sim 4{\cdot}3$ signal in tumour tissue is believed to be due to iron-transferrin, as in blood, and may be indicative of the presence of serum due to an inflammatory response. This response appears to be maintained in the degenerating region, while in the more viable region the signal falls, possibly indicating suppression of the response. MDMS treatment will have a greater effect on viable than on degenerating cells and this is reflected in the greater increase of the $g \sim 4{\cdot}3$ signal in the viable regions of the tumour.

Changes in the broad $g \sim 2$ signal are partially masked by those of other signals, and further work is required to separate the contributions of individual components. There is probably some contribution from the $g \sim 2{\cdot}05$ signal of ceruloplasmin. This would be expected to accompany the increase in iron-transferrin in the inflammatory response. The presence of serum in the inflammatory exudate is reflected in the ESR spectrum, which shows both iron-transferrin and ceruloplasmin signals as major components. However, in the tumour, changes in the iron-transferrin signal are not paralleled by those in the broad $g \sim 2$ signal. This, like the $g \sim 6$ signal, is probably associated with cellular degeneration.

### *Blood*

The changes observed in the spectrum of blood from Yoshida-bearing rats give an indication of the stage of the disease and differ markedly from those observed with blood from leukaemic mice (Dodd, 1975).

Ceruloplasmin and iron-transferrin remain constant until vascularization of the tumour implant. They then change in opposite directions. The decrease in iron-transferrin may result from a demand for iron by the tumour, and the increase in ceruloplasmin may be an attempt to restore the level of iron in the blood. The initial increase in iron-transferrin in the tumour implant appears to be insufficient to influence the level detectable in the blood. In the phase of generalized disease, when metastases are present, iron-transferrin increases and ceruloplasmin decreases. This probably reflects a breakdown in normal metabolism. In control experiments, it was shown that the changes in blood shortly after injection of MDMS are independent of the presence of the tumour. However, subsequent regression is marked by a return of the ceruloplasmin and iron-transferrin levels to normal.

*Spleen*

The g $\sim$ 4·3 signal in spleen represents a species different from that in tumour tissue. Its steady decrease may indicate loss of iron from the spleen to the developing tumour. The ascorbyl radical concentration of spleen tends to decrease (Silcock and Dodd, 1976), but the magnitude of the decrease is smaller than that of the g $\sim$ 6 signal.

*Lymph nodes*

Since the ESR spectra of lymph nodes, in the later stages of the disease, closely resemble those of the viable region of the tumour, they demonstrate the presence of malignant cells. The iron-transferrin signal of the nodes is reduced on invasion, but is restored after regression of the metastases. However, a change of cell type in the node does not affect the g $\sim$ 1·94 signal, suggesting that the overall mitochondrial activity is unaltered. The sudden increase in the g $\sim$ 6 signal, following MDMS treatment, is probably due to destruction of tumour cells resulting in release of haem iron in the ferric form, as is observed in the tumour, although not reflected in the blood.

### CONCLUSION

Detailed examination of some tissues associated with developing and regressing Yoshida tumours, by means of ESR, shows distinct changes in the metabolism of these tissues. These can act as a guide to the stage of the disease, although their exact nature is still unclear. Some may be involved in iron transport, as postulated for tissues of leukaemic mice, and/or immunological or general inflammatory reactions. The interrelationship of these changes is complex and appears to be different for different tissues. This may be a function of the site of malignancy and the nature of the involvement of particular tissues.

The authors are deeply indebted to Dr B. W. Fox for his advice during the course of the work and preparation of the manuscript, and for his generous gift of specially purified MDMS. They also thank Dr M. Ebert for helpful discussion and Mr R. Thompson for his skilful assistance in transplanting the tumours. The work was supported by the Medical Research Council and the Cancer Research Campaign. One of the authors (J. M. S.) was holder of an MRC Postgraduate Training Award.

### REFERENCES

Brennan, M. J., Cole, T. & Singley, J. A. (1966) A Unique Hyperfine ESR Spectrum in Mouse Neoplasms Analysed by Computer Simulation. *Proc. Soc. exp. Biol. Med.*, **123**, 715.

Dodd, N. J. F. (1973) Some EPR Signals in Tumour Tissue. *Br. J. Cancer*, **28**, 257.

Dodd, N. J. F. (1975) Electron Spin Resonance Study of Changes during the Development of a Mouse Myeloid Leukaemia. I. Paramagnetic Metal Ions. *Br. J. Cancer*, **32**, 108.

Duchesne, J. & van de Vorst, A. (1970) Free Radicals in Normal and Pathological Surviving Tissues. *Bull. Acad. r. Belg. Cl. Sci.*, **56**, 433.

Emanuel, N. M., Saprin, A. N., Shabalkin, V. A., Kozlova, L. E. & Kruglyakova, K. E. (1969) Detection and Investigation of a New Type of ESR Signal Characteristic of Some Tumour Tissues. *Nature, Lond.*, **222**, 165.

FOSTER, M. A., POCKLINGTON, T., MILLER, J. D. B. & MALLARD, J. R. (1973) A Study of Electron Spin Resonance Spectra of Whole Blood from Normal and Tumour Bearing Patients. *Br. J. Cancer*, **28**, 340.

FOX, B. W. (1969) The Sensitivity of a Yoshida Sarcoma to Methylene Dimethane Sulphonate. *Int. J. Cancer*, **4**, 54.

HOLLOCHER, T. C., SOLOMON, F. & RAGLAND, T. E. (1966) A Superfine Interaction Involving $^{33}$S in the Iron-containing Proteins of Azobacter vinelandii. *J. biol. Chem.*, **241**, 3452.

MAILER, C., SWARTZ, H. M., KONIECZNY, M., AMBEGAONKAR, S. & MOORE, V. L. (1974) Identity of the Paramagnetic Element found in Increased Concentrations in Plasma of Cancer Patients and its Relationship to other Pathological Processes. *Cancer Res.*, **34**, 637.

MALLARD, J. R. & KENT, M. (1969) Electron Spin Resonance in Biological Tissues. *Physics Med. Biol.*, **14**, 373.

MARUYAMA, T., KATAOKA, N., NAGASE, S., NAKADA, H., SATO, H. & SASAKI, H. (1971) Identification of Three Line Electron Spin Resonance Signal and its Relationships to Ascites Tumours. *Cancer Res.*, **31**, 179.

NEBERT, D. W. & MASON, H. S. (1963) An Electron Spin Resonance Study of Neoplasms. *Cancer Res.*, **23**, 833.

SILCOCK, J. M. & DODD, N. J. F. (1976) Electron Spin Resonance Study of Changes during Development of Solid Yoshida Tumour. I. Ascorbyl Radical. *Br. J. Cancer*, **34**, 550.

SLATER, T. F. & COOK, J. W. R. (1969) Electron Spin Resonance Studies on Normal and Malignant Human Tissues and in Normal and Damaged Rat Liver. In *Cytology Automation*. Ed. D. M. D. Evans. Edinburgh: Livingstone. p. 108.

SWARTZ, H. M. & WIESNER, J. (1972) Radiation Effects on Plasma Electron Spin Resonance (ESR) Spectra of Cancer Patients. *Radiology*, **104**, 209.

SWARTZ, H. M., MAILER, C., AMBEGAONKAR, S., ANTHOLINE, W. E., MCNELLIS, D. R. & SCHNELLER, S. J. (1973) Paramagnetic Changes during Development of a Transplanted AKR/J Leukemia in Mice as Measured by Electron Spin Resonance. *Cancer Res.*, **33**, 2588.

VITHAYATHIL, A. J., TERNBERG, J. L. & COMMONER, B. (1965) Changes in Electron Spin Resonance Signals of Rat Liver during Chemical Carcinogenesis. *Nature, Lond.*, **207**, 1246.